# Enhancer alterations in cancer: a source for a cell identity crisis

**DOI:** 10.1186/s13073-014-0077-3

**Published:** 2014-09-23

**Authors:** Ken J Kron, Swneke D Bailey, Mathieu Lupien

**Affiliations:** The Princess Margaret Cancer Centre – University Health Network, Toronto, ON M5G 1 L7 Canada; Department of Medical Biophysics, University of Toronto, Toronto, ON M5G 1 L7 Canada; Ontario Institute for Cancer Research, Toronto, ON M5G 0A3 Canada

## Abstract

Enhancers are selectively utilized to orchestrate gene expression programs that first govern pluripotency and then proceed to highly specialized programs required for the process of cellular differentiation. Whereas gene-proximal promoters are typically active across numerous cell types, distal enhancer activation is cell-type-specific and central to cell fate determination, thereby accounting for cell identity. Recent studies have highlighted the diversity of enhancer usage, cataloguing millions of such elements in the human genome. The disruption of enhancer activity, through genetic or epigenetic alterations, can impact cell-type-specific functions, resulting in a wide range of pathologies. In cancer, these alterations can promote a ‘cell identity crisis’, in which enhancers associated with oncogenes and multipotentiality are activated, while those promoting cell fate commitment are inactivated. Overall, these alterations favor an undifferentiated cellular phenotype. Here, we review the current knowledge regarding the role of enhancers in normal cell function, and discuss how genetic and epigenetic changes in enhancer elements potentiate oncogenesis. In addition, we discuss how understanding the mechanisms regulating enhancer activity can inform therapeutic opportunities in cancer cells and highlight key challenges that remain in understanding enhancer biology as it relates to oncology.

## Introduction

The development of cell identity during the differentiation process in multicellular organisms creates highly specialized cells and tissues that perform unique tasks. With the premise that the vast majority of cells in a multicellular organism contain the exact same genetic information, each distinct specialized cell has enhancers that are either active or inactive. Promoters, unlike enhancers, exist immediately adjacent to a gene, show directionality and tend to have a greater degree of overlapping activity across cell types compared with enhancers [1,2]. In humans, enhancers outnumber promoters and genes by approximately one order of magnitude [3,4] and their differential usage leads to diverse gene expression patterns, which allow for the creation of hundreds of cell functions and identities. In undifferentiated and pluripotent embryonic stem cells (ESCs), active enhancers are found in proximity to and drive the expression of genes involved in maintaining pluripotency [5], while genes involved in promoting lineage specification are surrounded by largely inactive enhancer elements [6]. During cellular differentiation, enhancers that control the expression of genes involved in lineage specification become active. For example, ESCs that are induced to differentiate into neuroectoderm cells gain enhancer activity surrounding genes specifically expressed in the neuroectoderm and show reduced activity of enhancers surrounding pluripotency-related genes [5,6]. The combinatorial binding of cell-type-specifying transcription factors (TFs) and epigenetic modifications drives this enhancer activity.

The loss of cell fate commitment and gain in pluripotency are central features of carcinogenesis [7-9]. Whole-genome sequencing approaches have provided evidence that enhancers are prime targets for genetic or epigenetic alterations that favor cancer development. From a genetics standpoint, these alterations include mutations to genes that encode chromatin looping factors and TFs, which act together to bring enhancers in close physical proximity with gene promoters in order to drive gene expression. In addition, genetic alterations can affect the enhancers themselves. Epigenetic changes include abnormal deposition or removal of histone modifications or DNA methylation that serve to activate enhancers that are normally repressed, or vice versa. The characterization of changes in enhancers occurring during tumor development and progression is delineating new therapeutic opportunities in the form of targeted epigenetic treatments and biomarker discovery.

In this review, we discuss enhancer biology as it pertains to the promotion of cell identity and we highlight recent findings demonstrating that genetic and epigenetic alterations influencing enhancer function are favorable to cancer development and progression. To conclude, we discuss the potential for treating cancers based on enhancer alterations and the need to address access to quality patient-derived samples and to delineate intratumor differential enhancer usage.

## Enhancers and cell identity

Enhancers define cell identity by establishing cell-type-specific gene transcription programs through the recruitment of TFs active in unique cell type(s) and through physical interactions with target gene promoters [10]. Enhancers can be discovered and defined based on a number of factors, including their epigenetic features, such as histone and DNA modifications, their transcription into non-coding RNAs, the proteins that bind them, and the three-dimensional topology that they promote. Below, we discuss each of these features and how they uniquely contribute to enhancer functionality in driving cell identities.

### The unique chromatin features of enhancers

Unlike promoters, which lie immediately upstream of the genes they regulate, enhancers can reside anywhere across the genome, including within intragenic regions [1,11-14]. Therefore, enhancer discovery presents a unique challenge. In recent years, the genome-wide mapping of epigenetic modifications that are specifically enriched at enhancers has greatly aided in their identification. For example, monomethylation and dimethylation of histone H3 on lysine 4 (H3K4me1/2) typify enhancers within a given cell type, although the H3K4me2 mark is also present at proximal promoter regions, albeit at weaker levels [1,15,16]. The additional presence of acetylated histones, such as H3 on lysine 27 (H3K27ac), is typical of active regulatory elements including enhancers. ‘Poised’ or inactive enhancers are similarly marked by H3K4me1/2, but are more likely to associate with histone H3 lysine 27 or lysine 9 di- or trimethylation [6,15,17]. DNA methylation at CpG dinucleotides can also mark inactive enhancers [18].

Mapping regions of open chromatin is another way to identify enhancers, and other regulatory elements, across the genome of any cell type. This can be accomplished through DNase I hypersensitive sites sequencing (DNase-seq), formaldehyde-assisted isolation of regulatory elements sequencing (FAIRE-seq) or assay for transposase-accessible chromatin sequencing (ATAC-seq) assays [4,19-21]. A third approach to mapping enhancers relies on the observation that active enhancers are bidirectionally transcribed into RNA, generating unique non-coding enhancer RNAs (eRNAs) [22-25].

The annotation of enhancers using these techniques has greatly propelled our understanding of enhancer biology as it relates to cell identity determination. For example, Stergachis *et al*. [26] used DNase-seq to show that, in addition to dramatic remodeling in which a number of gains and losses are observed, there is an overall net loss of regulatory elements when pluripotent ESCs are compared to more differentiated hematopoietic progenitors or to fully differentiated cells of the hematopoietic lineage. Similar results were also reported along the cardiac differentiation lineage [26]. Using TF DNA recognition motifs analysis within DNase I hypersensitive sites (DHSs), Stergachis *et al*. also showed a reduction in the total number of regulatory elements containing motifs for lineage-specific TFs [26]. For example, hematopoietic progenitor cells differentiating into B cells have fewer DHSs with the DNA recognition motif that is recognized by the natural killer-specific NFIL3 TF. In contrast, no reduction in this motif was reported during natural killer cell differentiation. Thus, progenitor cells maintain accessible enhancers and during differentiation undergo a reduction in the number of accessible enhancers that are unnecessary for the differentiation cell type.

The genome-wide annotation of enhancers reveals their diversity. In addition to the typical enhancers, low and highly occupied targets (LOTs/HOTs) and super/stretch enhancers reminiscent of the previously reported clusters of open regulatory elements (COREs) [20] have been reported. Super/stretch enhancers are of interest in cell fate determination because they preferentially exist proximal to cell-type-specific genes and recruit master regulatory TFs [5,27]. For example, murine ESC-specific super/stretch enhancers are bound by high levels of KLF4 and ESRRB, two critical factors for the pluripotency program, and surround genes that also contribute to pluripotency [5]. Super/stretch enhancers in B cells are bound by the PU.1 TF and map close to genes expressed in B cells, including *FOXO1* and *INPP5D* [5]. The recent annotation of super/stretch enhancers in 86 human cell and tissue types further showcases their relevance to cell identity [28].

### Enhancers serve as docking sites for proteins recruited by non-coding RNAs

Enhancer activity relies on binding TFs [29]. The human genome is believed to encode more than a thousand TFs [30]. These TFs bind enhancers by recognizing specific short DNA sequences (known as DNA recognition motifs) that lie in ‘open’ chromatin, which is characterized by reduced nucleosome occupancy [17,31]. To date, fewer than 200 DNA recognition motifs have been identified [30,32] and genome-wide binding profiles (cistromes) for a few hundred TFs are available [3,33,34]. While some TFs are required across many or most cell types, others appear to be lineage-specific [35]. For instance, PU.1 is found in the hematopoietic lineage and is necessary for B-cell differentiation [36]. Similarly, GATA1 is required in the hematopoietic lineage to promote erythroid differentiation [37].

In addition to TFs, enhancers can recruit additional factors to ensure their function. The specific epigenetic modifications found at enhancers are derived from the recruitment of epigenetic writers and erasers. For instance, the myeloid/lymphoid or mixed-lineage leukemia methylases MLL2, MLL3 and MLL4 (also known as KMT2D, KMT2C and KMT2B, respectively) are histone methyltransferases that bind regulatory elements and are responsible for deposition of the enhancer marks H3K4me1 and H3K4me2 [38-40]. Similarly, lysine acetyl transferases such as CBP (also known as CREBBP) and P300 (also known as EP300) bind enhancers to increase their activity through protein acetylation, inclusive of histones [41,42]. The EZH2 methyltransferase creates silenced or poised enhancers through the H3K27me3 modification [6,43]. DNA methylation also marks some silent enhancers in normal cells [44,45], with the DNA cytosine-5-methyltransferases DNMT1, DNMT3A and DNMT3B establishing this mark, and the TET methylcytosine dioxygenases TET1, TET2, TET3 necessary for active removal of DNA methylation [46,47]. The presence of specific epigenetic modifications at enhancers allows the recruitment of epigenetic readers. For example, BRD4 recognizes histone acetylation, including H3K27ac, leading it to occupy chromatin preferentially at cell-specific super/stretch enhancers [28,48,49].

Long non-coding RNAs (lncRNAs), which are RNAs of more than 200 nucleotides in length that lack protein-coding potential [50], can also serve as enhancer-like elements to regulate gene expression [51]. For instance, non-coding RNA-activating (ncRNA-a) regulates the expression of adjacent protein-coding genes independently of their orientation, similar to typical enhancer elements [51]. Other lncRNAs influence enhancer activity through their interaction with epigenetic factors. For example, the lncRNA HOTAIR interacts with the polycomb repressive complex 2 (PRC2) to facilitate the deposition of the H3K27me3 repressive epigenetic modification on the chromatin at the *HOXD* locus, whereas the lncRNA HOTTIP activates transcription of 5’ *HOXA* genes through recruitment of WDR5/MLL complexes [52]. Accordingly, lncRNAs can play a crucial role in maintaining cell identity. For example, the lncRNA Tcl1-upstream neural differentiation-associated RNA (TUNAR) interacts with a complex of proteins to promote expression of the pluripotency factors Sox2, Nanog and Fgf4 in mouse ESCs [53]. In addition, RNA-interference-mediated knockdown of seven abundantly expressed lncRNAs in mouse erythroid cells inhibited terminal erythroid differentiation [54], raising the possibility that a large number of tissue-specific lncRNAs are necessary for cell identity programs.

### Enhancers form chromatin interactions with target promoters

Enhancers rarely regulate the expression of the most proximal gene [55]. In fact, they may be separated from their target promoter(s) over genomic distances that can exceed millions of base pairs (megabases) [56,57]. Although enhancers can help recruit RNA polymerase II, which then tracks along the DNA to find its target promoter [58], enhancers are typically reported to act by physically interacting with their target gene promoters through long-range chromatin interactions, or loops [59,60]. These interactions form during cell differentiation [59,61] and are involved in establishing the chromatin architecture permissive to stimulus-specific transcriptional responses [62]. As enhancer usage is largely cell-type-specific, it is not surprising that these interactions are also unique to different cell types and undergo large-scale changes during differentiation [63].

Ubiquitously expressed proteins, including the CCCTC-binding factor (CTCF), as well as the cohesin and mediator complexes, are known to mediate chromatin interactions [59,64-68]. Chromatin immunoprecipitation coupled with next generation sequencing (ChIP-seq) assays against subunits of the cohesin complex, including SMC1A and SMC3, reveal that they localize to enhancers, promoters, regions bound by the mediator complex and cell-type-specific TFs [59,69]. The cohesin complex was also shown to mediate chromatin interactions, inclusive of those connecting promoters to enhancers [67,68]. CTCF has historically been associated with an insulator function in the genome, by which it blocks interactions between enhancers and promoters [70,71]. However, genome-wide profiling of CTCF binding and subunits of the cohesin complex exposed a substantial degree of overlap between these factors [72,73]. In addition, CTCF was observed to localize at tissue-specific enhancer elements [74,75], suggesting a role for CTCF in mediating physical interactions between DNA regulatory elements and in driving the chromosomal conformation that is necessary for cell type specification.

A role for eRNAs in promotion of long-range promoter-enhancer interactions to regulate gene expression has also been recently reported. Li *et al*. have described eRNAs induced by estrogen in breast cancer cells that mediate promoter-enhancer interactions that are also dependent on the cohesin complex [24]. Furthermore, Hsieh *et al*. also discovered an eRNA proximal to *KLK3* in the *KLK* locus that enables enhancer interactions with *KLK2* [76]. Others, however, have shown that inhibition of eRNA generation during the estrogen response in MCF7 breast cancer cells does not affect TF binding, epigenetic modifications or chromatin loop formation to target genes [77], suggesting that these events precede eRNA transcription.

## Enhancers and cancer

Enhancers provide a basis for cell identity. Thus, the maintenance of cell-type-specific enhancer activation is critical in order to avoid improper, or the lack of a necessary, enhancer function and the development of life-threatening malignancies. Indeed, recent whole-genome sequencing studies have established that alterations to enhancers can occur through aberrant epigenetic modifications, sequence variation, or mutations, within enhancer binding factors and within enhancers themselves. In this section we discuss the epigenetic and genetic changes that alter enhancer function and contribute to an altered cell identity.

### Epigenetic alterations affecting enhancer function in cancer

**Figure 1:**
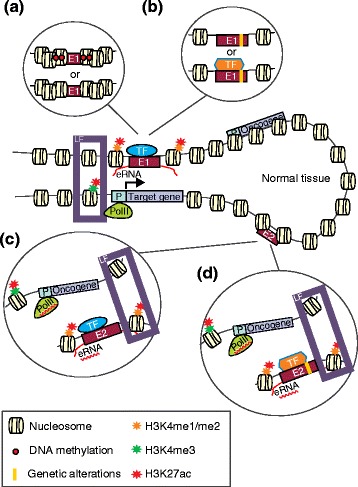
**Enhancer biology in normal and malignant cells.** The center of the figure shows how, in normal tissue, cell-type-specific transcription factors (TFs) bind to enhancer elements to drive expression of target cell identity genes, while enhancers utilized in alternative cell lineages are poised or silenced. **(a)** Enhancer (E1) repression in the course of cancer development through either acquisition of DNA methylation or chromatin compaction blocking TF binding. **(b)** Genetic alterations in an enhancer (E1), altering its normal function through either blocking TF binding or inducing the binding of a new TF. **(c)** Enhancer (E2) activation in the course of cancer development through epigenetic changes, resulting in chromatin openness favorable to TF binding and target gene expression. **(d)** Genetic alterations resulting in the activation of an enhancer (E2) normally inactive in normal cells. eRNA, enhancer RNA; LF, chromatin looping factors; P, promoter; PolII, RNA polymerase II.

Fluctuations in DNA methylation levels are typical of cancer development and can directly impact enhancer activity (Figure 1a, Table 1). Yegnasubramanian *et al*. described DNA methylation gains at conserved intergenic regions across chromosomes 21 and 22 in prostate cancer cells [78], indicating the potential for a previously unappreciated role of DNA hypermethylation in enhancer regions. Subsequently, Aran *et al*. further established DNA methylation changes in enhancer regions linked to cancer genes in diverse cell types including breast, lung and cervical cancer cell lines [79]. In addition, Taberlay *et al*. have recently described widespread changes in DNA methylation of nucleosome-depleted regions within distal regulatory elements in breast and prostate cancer cells [80]. Intriguingly, they found that the majority of epigenetic changes at enhancers from both benign and cancerous cells were gains in epigenetic silencing as opposed to aberrant activation, suggesting that it is a net loss of features that drives specific cell identity.

**Table 1:** **Epigenetic alterations of enhancers found in malignancies**

**Cancer type(s)**	**Epigenetic change**	**Reference(s)**
Colon	Gains and losses of H3K4me1	[81]
Breast, cervical, colon, pancreatic, prostate, blood	Gains and losses of super-enhancers	[28,82,83]
Breast, lung, cervical	Gains and losses of DNA methylation	[79]
Melanoma, breast, T-cell leukemia	Gains and losses of open chromatin (DNase)	[26]
Breast	Gains and losses of H3K4me2, open chromatin	[84]
T-cell acute lymphoblastic leukemia	Global chromatin compaction, reduced H3K27ac	[85]
Breast, colon, laryngeal squamous cell	HOTAIR overexpression, gains of H3K27me3	[86-88]
Hepatocellular	HOTTIP overexpression, increased HOXA13 overexpression	[89]
Colon	*CCAT1-L* overexpression, chromatin looping to *MYC*	[90,91]

Abnormal patterns of histone modifications at enhancers also characterize the development and progression of several malignancies (Figure 1a,b, Table 1). Through H3K4me1 ChIP-seq assays, Akhtar-Zaidi *et al*. [81] identified differential enhancer usage between normal and primary colorectal tumor cells, terming these ‘variant enhancer loci’ (VELs). VELs were found to correlate with the transcription of putative target genes and predicted gene expression patterns in a manner that was concordant with a gain or loss of enhancer state [81]. In addition, there was a markedly greater repression of genes associated with enhancer loss than there was activation of genes associated with enhancer gain. Gains in enhancer activity were also reported at loci associated with ESCs during cancer development and progression [26]. Using DNase-seq data to define active regulatory elements based on chromatin openness, followed by principle component analysis, Stergachis *et al*. determined that tumorigenic cells commonly displayed a regulatory landscape more similar to that of ESCs as opposed to differentiated cells of varying origin (that is, endoderm, ectoderm, mesoderm) [26]. In addition, gains of open chromatin were observed in other cell lineages and in sites not observed in any normal cells, suggesting that cancer cells invoke the activity of atypical enhancers to activate oncogenic pathways [26].

With respect to tumor progression, Magnani *et al*. described distinct epigenetic landscapes associated with enhancers in breast cancer cells resistant to endocrine therapy compared with those responsive to treatment [84]. This revealed that endocrine-therapy-resistant cells rely on the NOTCH signaling pathway to elicit alternative enhancer usage and cell survival independent of estrogen signaling [84]. Active NOTCH signaling is required for normal mammary stem cell function [92], implying that the development of endocrine therapy resistance in breast cancer cells may rely on the reversion or reactivation of stemness pathways and a loss of estrogen responsiveness that is typical of luminal breast cancer cell identity.

VELs are not restricted to single enhancers but can also give rise to super/stretch enhancers. This has been documented in diverse cancer types, including multiple myeloma, B-cell lymphoma, colon, prostate, breast and cervical cancers [28,82,83]. Specific genes, such as the *MYC* oncogene, are associated with variant enhancer loci that cluster with other VELs to form super/stretch VELs in many cancer types [28]. These are reminiscent of super/stretch enhancers. Other genes preferentially associate with super-VELs only in specific cancer types, such as *XBP1* in multiple myeloma, in which it is known to be critical for disease development [93,94].

### Genetic alterations modulate enhancer function in cancer

Enhancers are also hotspots of genetic alterations promoting cancer development. The majority of disease-associated single nucleotide polymorphisms (SNPs) and their associated loci commonly lie within non-coding regions of the genome and thus do not directly alter the amino acid sequence of a protein [95]. These disease-associated SNPs typically map to enhancers [95-98] and directly alter the binding affinity of TFs for their respective DNA recognition motifs (Figure 1c,d) [57,96,99-102]. For example, breast-cancer-associated SNPs map to enhancers bound by the forkhead box A1 (FOXA1) TF and ERα, and modulate the affinity of FOXA1 for DNA, resulting in altered target gene expression [96]. Similar mechanisms are at play in prostate cancer [57,99,103], colon cancer [81] and acute myeloid leukemia (AML) [104]. Enhancers targeted by risk variants associated with colorectal cancer are significantly enriched within VELs for this disease [81]. Similarly, SNPs associated with breast cancer are significantly enriched within differentially methylated enhancer elements in breast cancer [105]. This suggests a convergence on these enhancers, by which their activity can be altered, either through genetic or epigenetic alterations.

Whole-genome sequencing of tumor samples has identified thousands of somatic mutations outside of coding regions [106-108]. While only a subset of these mutations is likely driving cancer development, the fact that enhancers can be affected by mutations that predispose to cancer supports the idea that somatic mutations may alter enhancer function (Figure 1c,d, Table 2). Several lines of evidence support this hypothesis. For example, translocations commonly found in Burkitt’s lymphoma place the *MYC* oncogene in control of intronic and 3’ *IGH* enhancers, ultimately leading to deregulated expression of *MYC* and the development of lymphoma [109,110]. Deletions of the locus control region that contains enhancers controlling β-globin gene expression have also been described in sickle-cell anemia patients [111]. Point mutations within the telomerase reverse transcriptase (*TERT*) promoter enhance *TERT* expression in melanoma, and in cancers of the central nervous system, bladder and thyroid [112-116]. These mutations provide *de novo* DNA binding motifs for ETS family TFs [114]. Finally, point mutations in enhancers surrounding *SHH* and *SOX9* lead to polydactyly and a form of severe skeletal malformation (campomelic dysplasia), respectively [117,118], while point mutations in enhancers proximal to *TBX5* and *PTF1A* lead to congenital heart defects and pancreatic agenesis, respectively [119,120].

**Table 2:** **Mutations found in factors associated with enhancer function**

**Cancer type(s)**	**Mutation**	**Reference(s)**
Burkitt’s lymphoma	*IGH/MYC* translocation	[109]
Melanoma, central nervous system, bladder, thyroid	*TERT* promoter	[112-116]
Breast, prostate	FOXA1	[121,122]
AML, myelodysplastic syndromes	GATA2	[123]
Breast, lung	GATA3	[122,124-127]
AML, breast, urothelial	RUNX1	[122,127,128]
Bladder, head and neck, lung, urothelial, breast	MLL2/MLL3/MLL4	[122,127,129]
B-cell lymphoma, lung	EZH2	[122,127,130]
AML, lung	DNMT3A	[122,127,131]
AML, bladder, lung, urothelial	TET2	[122,127,132]
Urothelial, bladder, breast, head and neck	CTCF	[122,127,133]
Bladder, glioblastoma, lung, urothelial	STAG2	[122,127,134]
Bladder, urothelial, AML	SMC1A	[122,127,135]
Bladder, AML, lung	SMC3	[122,127,135]
Lung, AML	RAD21	[122,127]
Transitional cell carcinoma	NIPBL	[135]
Prostate, adrenocortical, uterine leiomyoma	MED12	[121,127,136,137]

AML, acute myeloid leukemia.

### Genetic alterations in enhancer-associated factors

Tumor sequencing efforts have identified mutations in genes encoding lineage-specific TFs that preferentially bind enhancers, such as *FOXA1* and members of the GATA binding protein family (Table 2) [62,121-124,138,139]. *FOXA1* mutations have been discovered in breast and prostate cancers [121]. These mutations occur within the DNA binding and C-terminal domains of the protein and a subset was shown to be favorable to tumor growth [62]. GATA2 is a TF that is critical for the formation of primitive erythroid cells and is expressed in hematopoetic stem and progenitor cells [139,140], while GATA3 plays an important role in luminal differentiation of breast epithelial cells [141]. Mutations in *GATA2* are prevalent in familial AML/myelodysplastic syndromes [123], whereas mutations of GATA3 occur in ~10% of breast cancers [124-126]. RUNX1 (a TF required for differentiation of blood cells) is another example of a lineage-specific TF that is preferentially mutated in AML (~9% of cases) compared with other cancer types (mutated in less than 4% of cases for other cancer types) [122].

Mutations in epigenetic factors that bind enhancers have also been reported in cancer. For example, the enzymes responsible for the H3K4me1/me2 epigenetic modifications, specifically the *MLL2*, *MLL3* and *MLL4* genes, are significantly mutated in three or more cancer types [122,127,129]. EZH2 is also commonly mutated in diffuse large B-cell lymphomas and follicular lymphomas [130]. Although DNA methylation is not uniquely found at enhancers, mutations in the *DNMT3A* and *TET2* genes were reported in AML [131,132]. DNMT3A is a methyltransferase involved in the *de novo* methylation of CpG dinucleotides [86] and TET2 converts methylcytosine to 5-hydroxymethylcytosine [142].

Finally, factors involved in long-range chromatin interactions, including *CTCF*, the cohesin subunit stromal antigen 2 (*STAG2*) [122,127], *SMC1A*, *SMC3*, *RAD21* and the loading protein Nipped-B-like (*NIPBL*) are significantly mutated in cancer [134,135]. Furthermore, the mediator complex subunit *MED12*, which is known to contribute to chromatin loop formation [59], is mutated in cancers of the prostate and adrenal cortex [121,136]. The exact role of these mutations remains to be clarified, but the idea that they could provide an oncogenic benefit by affecting chromatin interactions, and thus modifying enhancer-promoter interactions, warrants further investigation. Taken together, these results suggest that mutations in lineage-specific TFs, epigenetic enzymes and chromatin-interaction factors can promote cancer development. Whether these genetic changes impinge upon TF binding, epigenetic profiles or overall chromatin conformation, and whether this has an effect on cell identity is not known and should be the focus of future research investigating specific mutations.

Deregulated expression of lncRNAs that can impinge on enhancer activity may also contribute to tumorigenesis. For example, HOTAIR overexpression in breast cancer leads to genome-wide alterations in H3K27me3 and promotes invasive and metastatic cell properties [143]. HOTTIP is also overexpressed in hepatocellular carcinoma, leading to increased HOXA13 expression and cell proliferation [89]. Furthermore, colon-cancer-associated transcript 1-long isoform (CCAT1-L) lncRNA is found in a super-enhancer upstream of the *MYC* oncogene, where it promotes looping and expression of MYC [90]. Yang *et al*. have also studied two prostate-cancer-associated lncRNAs, PRNCR1 and PCGEM1, characterizing them as interacting with the androgen receptor (AR) TF, facilitating the looping of AR-bound enhancers [144]. These lncRNAs also promoted ligand-independent activation of the AR transcriptional program, thereby potentially contributing to castration-resistant prostate cancer development. However, Prensner *et al*., using RNA immunoprecipitation studies, failed to detect the interaction between these lncRNAs and AR, questioning the validity of the initial findings [145]. While it remains plausible that lncRNAs act as co-factors in TF-enhancer interactions that promote cancer progression, additional work is needed to address these discordant results.

### Implications for medicine

Alterations in enhancer usage and activity are a driving force behind oncogenesis and thus have broad medical applications. First, both genetic and epigenetic changes in enhancers may be useful as biomarkers for both diagnosis and prognosis of cancer. SNP profiles, for example, can distinguish the relative likelihood of developing particular neoplasms. DNA methylation of enhancers may provide useful prognostic information beyond classical pathological parameters. The vast majority of work to date in the field of DNA methylation, however, has been heavily promoter biased. An enhancer focus may yield more clinical information.

Epigenetic modifications to enhancers are also therapeutically targetable, given the recent development of numerous inhibitors to epigenetic readers, writers and erasers. For example, bromodomain inhibitors are being widely investigated for their potential as anti-neoplastic agents. These compounds act by binding the bromodomain of the BET family of proteins, blocking their binding to acetyl-lysine residues and inhibiting activation of gene transcription. Interestingly, the unique features of super/stretch enhancers may make them more responsive than typical enhancers to such inhibitors. For instance, the treatment of myeloma cells with JQ1, a BET bromodomain inhibitor, decreases their proliferation with concomitant reductions in super-VEL-associated oncogene expression [82].

## Conclusions, future directions and perspectives

Enhancers are components of the genome that function to regulate gene expression and are critical for proper cellular differentiation. The identity of any given cell type is tied to the cell-type-specifying TFs that it expresses and, in turn, to the enhancers that these TFs bind. Abnormal enhancer activation or repression and TF activity drive cancer development and progression through the activation of oncogenes and expression programs from alternative cell lineages, in conjunction with the silencing of tumor suppressor genes and programs necessary for terminal differentiation. These enhancer alterations have the potential to be used both as markers of disease and as avenues for therapeutic intervention.

Whole-genome profiling strategies, particularly when based on massively parallel sequencing, have greatly increased the rate at which new discoveries are made regarding enhancer biology in both a normal- and tumor-cell setting. Projects such as the Encyclopedia of DNA Elements (ENCODE) have greatly expanded our knowledge of the functional genome beyond coding sequences [3]. Current efforts, such as those led by the Roadmap Epigenomics Program and the International Human Epigenome Consortium (IHEC) are geared towards characterizing the functional genome in human tissues [146,147]. Studies using cancer tissues as opposed to cell lines will also be necessary. Using tissue samples, however, will present a series of challenges, including cellular heterogeneity in bulk specimens [148]. Sorting cells using cell-type-specific markers followed by regulatory element profiling may overcome these challenges. As an example of this issue, breast epithelium consists of distinct epithelial cell types, and it is postulated that unique cell types give rise to the different breast cancer subtypes [149-152]. If true, it is likely that many enhancer alterations described in cancer are representative of a specific cell of origin present in only a fraction of normal breast cells. The heterogeneous mixture of cancer cells in tumors with differing capacities to proliferate, migrate and regenerate also poses a challenge when using tissue samples [148]. Identifying subpopulations of cancer cells with differential enhancer usage compared with the bulk may help to better characterize the biology behind aggressive and metastatic phenotypes.

Despite the challenges that lie ahead, we have gained a greater understanding of the role that enhancers play in tumor development and progression. Causal mutations in enhancers [109,110] and the gain of super/stretch enhancers driving oncogene expression [28,82,83] strongly support a role for enhancers in tumor development. The discovery and proven efficacy of new therapeutic agents that target epigenetic factors found at enhancers, such as JQ1, further substantiates the importance of enhancers in cancer. Future research focusing on the assignment of enhancers to their target genes may also have clinical implications when considering disease prognoses and targeted therapies. Genome-wide profiling of mutations that map to enhancers or of the activation or inactivation of enhancers in tumors is anticipated to reveal particular disease outcomes and point to therapies that can be tailored to the specific transcriptional network associated with these genetically and/or epigenetically altered enhancers in cancer.

Enhancers play a central role in cellular identity and increasing evidence demonstrates that they are primary targets of alterations promoting cancer development and progression. Overall, this strongly supports a transition in cancer research from the gene-centric view to a comprehensive approach inclusive of these non-coding regulatory elements.

## Abbreviations

AML: Acute myeloid leukemia
AR: Androgen receptor
ChIP-seq: Chromatin immunoprecipitation sequencing
DHSs: DNase I hypersensitive sites
DNase-seq: DNase I hypersensitive sites sequencing
eRNA: Enhancer RNA
ESC: Embryonic stem cell
H3K27ac: Histone H3 lysine 27 acetylation
H3K27me2/3: Histone H3 lysine 27 di/trimethylation
H3K4me1/2/3: Histone H3 lysine 4 mono/di/trimethylation
lncRNA: Long non-coding RNA
SNP: Single nucleotide polymorphism
TF: Transcription factor
VELs: Variant enhancer loci

## References

[1] Heintzman ND, Ren B (2009). Finding distal regulatory elements in the human genome. Curr Opin Genet Dev.

[2] Koch CM, Andrews RM, Flicek P, Dillon SC, Karaöz U, Clelland GK, Wilcox S, Beare DM, Fowler JC, Couttet P, James KD, Lefebvre GC, Bruce AW, Dovey OM, Ellis PD, Dhami P, Langford CF, Weng Z, Birney E, Carter NP, Vetrie D, Dunham I (2007). The landscape of histone modifications across 1% of the human genome in five human cell lines. Genome Res.

[3] Consortium EP, Bernstein BE, Birney E, Dunham I, Green ED, Gunter C, Snyder M (2012). An integrated encyclopedia of DNA elements in the human genome. Nature.

[4] Thurman RE, Rynes E, Humbert R, Vierstra J, Maurano MT, Haugen E, Sheffield NC, Stergachis AB, Wang H, Vernot B, Garg K, John S, Sandstrom R, Bates D, Boatman L, Canfield TK, Diegel M, Dunn D, Ebersol AK, Frum T, Giste E, Johnson AK, Johnson EM, Kutyavin T, Lajoie B, Lee BK, Lee K, London D, Lotakis D, Neph S (2012). The accessible chromatin landscape of the human genome. Nature.

[5] Whyte WA, Orlando DA, Hnisz D, Abraham BJ, Lin CY, Kagey MH, Rahl PB, Lee TI, Young RA (2013). Master transcription factors and mediator establish super-enhancers at key cell identity genes. Cell.

[6] Rada-Iglesias A, Bajpai R, Swigut T, Brugmann SA, Flynn RA, Wysocka J (2011). A unique chromatin signature uncovers early developmental enhancers in humans. Nature.

[7] Abate-Shen C (2002). Deregulated homeobox gene expression in cancer: cause or consequence?. Nat Rev Cancer.

[8] Schwede M, Spentzos D, Bentink S, Hofmann O, Haibe-Kains B, Harrington D, Quackenbush J, Culhane AC (2013). Stem cell-like gene expression in ovarian cancer predicts type II subtype and prognosis. PLoS One.

[9] Ben-Porath I, Thomson MW, Carey VJ, Ge R, Bell GW, Regev A, Weinberg RA (2008). An embryonic stem cell-like gene expression signature in poorly differentiated aggressive human tumors. Nat Genet.

[10] Nolis IK, McKay DJ, Mantouvalou E, Lomvardas S, Merika M, Thanos D (2009). Transcription factors mediate long-range enhancer-promoter interactions. Proc Natl Acad Sci U S A.

[11] Hyder SM, Nawaz Z, Chiappetta C, Yokoyama K, Stancel GM (1995). The protooncogene *c-jun* contains an unusual estrogen-inducible enhancer within the coding sequence. J Biol Chem.

[12] Ritter DI, Dong Z, Guo S, Chuang JH (2012). Transcriptional enhancers in protein-coding exons of vertebrate developmental genes. PLoS One.

[13] Levine M (2010). Transcriptional enhancers in animal development and evolution. Curr Biol.

[14] Smith E, Shilatifard A (2014). Enhancer biology and enhanceropathies. Nat Struct Mol Biol.

[15] Heintzman ND, Stuart RK, Hon G, Fu Y, Ching CW, Hawkins RD, Barrera LO, Van Calcar S, Qu C, Ching KA, Wang W, Weng Z, Green RD, Crawford GE, Ren B (2007). Distinct and predictive chromatin signatures of transcriptional promoters and enhancers in the human genome. Nat Genet.

[16] Lupien M, Eeckhoute J, Meyer CA, Wang Q, Zhang Y, Li W, Carroll JS, Liu XS, Brown M (2008). FoxA1 translates epigenetic signatures into enhancer-driven lineage-specific transcription. Cell.

[17] Creyghton MP, Cheng AW, Welstead GG, Kooistra T, Carey BW, Steine EJ, Hanna J, Lodato MA, Frampton GM, Sharp PA, Boyer LA, Young RA, Jaenisch R (2010). Histone H3K27ac separates active from poised enhancers and predicts developmental state. Proc Natl Acad Sci U S A.

[18] Wiench M, John S, Baek S, Johnson TA, Sung MH, Escobar T, Simmons CA, Pearce KH, Biddie SC, Sabo PJ, Thurman RE, Stamatoyannopoulos JA, Hager GL (2011). DNA methylation status predicts cell type-specific enhancer activity. EMBO J.

[19] Gross DS, Garrard WT (1988). Nuclease hypersensitive sites in chromatin. Ann Rev Biochem.

[20] Gaulton KJ, Nammo T, Pasquali L, Simon JM, Giresi PG, Fogarty MP, Panhuis TM, Mieczkowski P, Secchi A, Bosco D, Berney T, Montanya E, Mohlke KL, Lieb JD, Ferrer J (2010). A map of open chromatin in human pancreatic islets. Nat Genet.

[21] Buenrostro JD, Giresi PG, Zaba LC, Chang HY, Greenleaf WJ (2013). Transposition of native chromatin for fast and sensitive epigenomic profiling of open chromatin, DNA-binding proteins and nucleosome position. Nat Methods.

[22] Kim TK, Hemberg M, Gray JM, Costa AM, Bear DM, Wu J, Harmin DA, Laptewicz M, Barbara-Haley K, Kuersten S, Markenscoff-Papadimitriou E, Kuhl D, Bito H, Worley PF, Kreiman G, Greenberg ME (2010). Widespread transcription at neuronal activity-regulated enhancers. Nature.

[23] Koch F, Fenouil R, Gut M, Cauchy P, Albert TK, Zacarias-Cabeza J, Spicuglia S, de la Chapelle AL, Heidemann M, Hintermair C, Eick D, Gut I, Ferrier P, Andrau JC (2011). Transcription initiation platforms and GTF recruitment at tissue-specific enhancers and promoters. Nat Struct Mol Biol.

[24] Li W, Notani D, Ma Q, Tanasa B, Nunez E, Chen AY, Merkurjev D, Zhang J, Ohgi K, Song X, Oh S, Kim HS, Glass CK, Rosenfeld MG (2013). Functional roles of enhancer RNAs for oestrogen-dependent transcriptional activation. Nature.

[25] Andersson R, Gebhard C, Miguel-Escalada I, Hoof I, Bornholdt J, Boyd M, Chen Y, Zhao X, Schmidl C, Suzuki T, Ntini E, Arner E, Valen E, Li K, Schwarzfischer L, Glatz D, Raithel J, Lilje B, Rapin N, Bagger FO, Jørgensen M, Andersen PR, Bertin N, Rackham O, Burroughs AM, Baillie JK, Ishizu Y, Shimizu Y, Furuhata E, Maeda S (2014). An atlas of active enhancers across human cell types and tissues. Nature.

[26] Stergachis AB, Neph S, Reynolds A, Humbert R, Miller B, Paige SL, Vernot B, Cheng JB, Thurman RE, Sandstrom R, Haugen E, Heimfeld S, Murry CE, Akey JM, Stamatoyannopoulos JA (2013). Developmental fate and cellular maturity encoded in human regulatory DNA landscapes. Cell.

[27] Parker SC, Stitzel ML, Taylor DL, Orozco JM, Erdos MR, Akiyama JA, van Bueren KL, Chines PS, Narisu N, Black BL, Visel A, Pennacchio LA, Collins FS, NISC Comparative Sequencing Program (2013). Chromatin stretch enhancer states drive cell-specific gene regulation and harbor human disease risk variants. Proc Natl Acad Sci U S A.

[28] Hnisz D, Abraham BJ, Lee TI, Lau A, Saint-Andre V, Sigova AA, Hoke HA, Young RA (2013). Super-enhancers in the control of cell identity and disease. Cell.

[29] Spitz F, Furlong EE (2012). Transcription factors: from enhancer binding to developmental control. Nat Rev Genet.

[30] Vaquerizas JM, Kummerfeld SK, Teichmann SA, Luscombe NM (2009). A census of human transcription factors: function, expression and evolution. Nat Rev Genet.

[31] Gorisch SM, Wachsmuth M, Toth KF, Lichter P, Rippe K (2005). Histone acetylation increases chromatin accessibility. J Cell Sci.

[32] Wang J, Zhuang J, Iyer S, Lin X, Whitfield TW, Greven MC, Pierce BG, Dong X, Kundaje A, Cheng Y, Rando OJ, Birney E, Myers RM, Noble WS, Snyder M, Weng Z (2012). Sequence features and chromatin structure around the genomic regions bound by 119 human transcription factors. Genome Res.

[33] Tang Q, Chen Y, Meyer C, Geistlinger T, Lupien M, Wang Q, Liu T, Zhang Y, Brown M, Liu XS (2011). A comprehensive view of nuclear receptor cancer cistromes. Cancer Res.

[34] Liu T, Ortiz JA, Taing L, Meyer CA, Lee B, Zhang Y, Shin H, Wong SS, Ma J, Lei Y, Pape UJ, Poidinger M, Chen Y, Yeung K, Brown M, Turpaz Y, Liu XS (2011). Cistrome: an integrative platform for transcriptional regulation studies. Genome Biol.

[35] Neph S, Stergachis AB, Reynolds A, Sandstrom R, Borenstein E, Stamatoyannopoulos JA (2012). Circuitry and dynamics of human transcription factor regulatory networks. Cell.

[36] Mandel EM, Grosschedl R (2010). Transcription control of early B cell differentiation. Curr Opin Immunol.

[37] Pevny L, Simon MC, Robertson E, Klein WH, Tsai SF, D'Agati V, Orkin SH, Costantini F (1991). Erythroid differentiation in chimaeric mice blocked by a targeted mutation in the gene for transcription factor GATA-1. Nature.

[38] Kaikkonen MU, Spann NJ, Heinz S, Romanoski CE, Allison KA, Stender JD, Chun HB, Tough DF, Prinjha RK, Benner C, Glass CK (2013). Remodeling of the enhancer landscape during macrophage activation is coupled to enhancer transcription. Mol Cell.

[39] Hu D, Gao X, Morgan MA, Herz HM, Smith ER, Shilatifard A (2013). The MLL3/MLL4 branches of the COMPASS family function as major histone H3K4 monomethylases at enhancers. Mol Cell Biol.

[40] Cheng J, Blum R, Bowman C, Hu D, Shilatifard A, Shen S, Dynlacht BD (2014). A role for H3K4 monomethylation in gene repression and partitioning of chromatin readers. Mol Cell.

[41] Jin Q, Yu LR, Wang L, Zhang Z, Kasper LH, Lee JE, Wang C, Brindle PK, Dent SY, Ge K (2011). Distinct roles of GCN5/PCAF-mediated H3K9ac and CBP/p300-mediated H3K18/27 ac in nuclear receptor transactivation. EMBO J.

[42] Tie F, Banerjee R, Stratton CA, Prasad-Sinha J, Stepanik V, Zlobin A, Diaz MO, Scacheri PC, Harte PJ (2009). CBP-mediated acetylation of histone H3 lysine 27 antagonizes *Drosophila* Polycomb silencing. Development.

[43] Kuzmichev A, Nishioka K, Erdjument-Bromage H, Tempst P, Reinberg D (2002). Histone methyltransferase activity associated with a human multiprotein complex containing the Enhancer of Zeste protein. Genes Dev.

[44] Chamberlain AA, Lin M, Lister RL, Maslov AA, Wang Y, Suzuki M, Wu B, Greally JM, Zheng D, Zhou B (2014). DNA methylation is developmentally regulated for genes essential for cardiogenesis. J Am Heart Assoc.

[45] Ronnerblad M, Andersson R, Olofsson T, Douagi I, Karimi M, Lehmann S, Hoof I, De Hoon M, Itoh M, Nagao-Sato S, Kawaji H, Lassmann T, Carninci P, Hayashizaki Y, Forrest AR, Sandelin A, Ekwall K, Arner E, Lennartsson A, FANTOM consortium (2014). Analysis of the DNA methylome and transcriptome in granulopoiesis reveals timed changes and dynamic enhancer methylation. Blood.

[46] Turek-Plewa J, Jagodzinski PP (2005). The role of mammalian DNA methyltransferases in the regulation of gene expression. Cell Mol Biol Lett.

[47] Kohli RM, Zhang Y (2013). TET enzymes, TDG and the dynamics of DNA demethylation. Nature.

[48] Dey A, Chitsaz F, Abbasi A, Misteli T, Ozato K (2003). The double bromodomain protein Brd4 binds to acetylated chromatin during interphase and mitosis. Proc Natl Acad Sci U S A.

[49] Zhang W, Prakash C, Sum C, Gong Y, Li Y, Kwok JJ, Thiessen N, Pettersson S, Jones SJ, Knapp S, Yang H, Chin KC (2012). Bromodomain-containing protein 4 (BRD4) regulates RNA polymerase II serine 2 phosphorylation in human CD4^+^ T cells. J Biol Chem.

[50] Bergmann JH, Spector DL (2014). Long non-coding RNAs: modulators of nuclear structure and function. Curr Opin Cell Biol.

[51] Orom UA, Derrien T, Beringer M, Gumireddy K, Gardini A, Bussotti G, Lai F, Zytnicki M, Notredame C, Huang Q, Guigo R, Shiekhattar R (2010). Long noncoding RNAs with enhancer-like function in human cells. Cell.

[52] Wang KC, Yang YW, Liu B, Sanyal A, Corces-Zimmerman R, Chen Y, Lajoie BR, Protacio A, Flynn RA, Gupta RA, Wysocka J, Lei M, Dekker J, Helms JA, Chang HY (2011). A long noncoding RNA maintains active chromatin to coordinate homeotic gene expression. Nature.

[53] Lin N, Chang KY, Li Z, Gates K, Rana ZA, Dang J, Zhang D, Han T, Yang CS, Cunningham TJ, Head SR, Duester G, Dong PD, Rana TM (2014). An evolutionarily conserved long noncoding RNA TUNA controls pluripotency and neural lineage commitment. Mol Cell.

[54] Paralkar VR, Mishra T, Luan J, Yao Y, Kossenkov AV, Anderson SM, Dunagin M, Pimkin M, Gore M, Sun D, Konuthula N, Raj A, An X, Mohandas N, Bodine DM, Hardison RC, Weiss MJ (2014). Lineage and species-specific long noncoding RNAs during erythro-megakaryocytic development. Blood.

[55] Sanyal A, Lajoie BR, Jain G, Dekker J (2012). The long-range interaction landscape of gene promoters. Nature.

[56] Lettice LA, Heaney SJ, Purdie LA, Li L, de Beer P, Oostra BA, Goode D, Elgar G, Hill RE, de Graaff E (2003). A long-range Shh enhancer regulates expression in the developing limb and fin and is associated with preaxial polydactyly. Hum Mol Genet.

[57] Zhang X, Cowper-Sal lari R, Bailey SD, Moore JH, Lupien M (2012). Integrative functional genomics identifies an enhancer looping to the *SOX9* gene disrupted by the 17q24.3 prostate cancer risk locus. Genome Res.

[58] Wang Q, Carroll JS, Brown M (2005). Spatial and temporal recruitment of androgen receptor and its coactivators involves chromosomal looping and polymerase tracking. Mol Cell.

[59] Kagey MH, Newman JJ, Bilodeau S, Zhan Y, Orlando DA, van Berkum NL, Ebmeier CC, Goossens J, Rahl PB, Levine SS, Taatjes DJ, Dekker J, Young RA (2010). Mediator and cohesin connect gene expression and chromatin architecture. Nature.

[60] Dekker J, Rippe K, Dekker M, Kleckner N (2002). Capturing chromosome conformation. Science.

[61] Fraser J, Rousseau M, Shenker S, Ferraiuolo MA, Hayashizaki Y, Blanchette M, Dostie J (2009). Chromatin conformation signatures of cellular differentiation. Genome Biol.

[62] Jin HJ, Zhao JC, Ogden I, Bergan RC, Yu J (2013). Androgen receptor-independent function of FoxA1 in prostate cancer metastasis. Cancer Res.

[63] Zhang Y, Wong CH, Birnbaum RY, Li G, Favaro R, Ngan CY, Lim J, Tai E, Poh HM, Wong E, Mulawadi FH, Sung WK, Nicolis S, Ahituv N, Ruan Y, Wei CL (2013). Chromatin connectivity maps reveal dynamic promoter-enhancer long-range associations. Nature.

[64] Phillips-Cremins JE, Sauria ME, Sanyal A, Gerasimova TI, Lajoie BR, Bell JS, Ong CT, Hookway TA, Guo C, Sun Y, Bland MJ, Wagstaff W, Dalton S, McDevitt TC, Sen R, Dekker J, Taylor J, Corces VG (2013). Architectural protein subclasses shape 3D organization of genomes during lineage commitment. Cell.

[65] Faure AJ, Schmidt D, Watt S, Schwalie PC, Wilson MD, Xu H, Ramsay RG, Odom DT, Flicek P (2012). Cohesin regulates tissue-specific expression by stabilizing highly occupied *cis*-regulatory modules. Genome Res.

[66] Merkenschlager M, Odom DT (2013). CTCF and cohesin: linking gene regulatory elements with their targets. Cell.

[67] Hadjur S, Williams LM, Ryan NK, Cobb BS, Sexton T, Fraser P, Fisher AG, Merkenschlager M (2009). Cohesins form chromosomal *cis*-interactions at the developmentally regulated *IFNG* locus. Nature.

[68] Seitan VC, Hao B, Tachibana-Konwalski K, Lavagnolli T, Mira-Bontenbal H, Brown KE, Teng G, Carroll T, Terry A, Horan K, Marks H, Adams DJ, Schatz DG, Aragon L, Fisher AG, Krangel MS, Nasmyth K, Merkenschlager M (2011). A role for cohesin in T-cell-receptor rearrangement and thymocyte differentiation. Nature.

[69] Schmidt CK, Brookes N, Uhlmann F (2009). Conserved features of cohesin binding along fission yeast chromosomes. Genome Biol.

[70] Bell AC, West AG, Felsenfeld G (1999). The protein CTCF is required for the enhancer blocking activity of vertebrate insulators. Cell.

[71] Phillips JE, Corces VG (2009). CTCF: master weaver of the genome. Cell.

[72] Parelho V, Hadjur S, Spivakov M, Leleu M, Sauer S, Gregson HC, Jarmuz A, Canzonetta C, Webster Z, Webster Z, Nesterova T, Cobb BS, Yokomori K, Dillon N, Aragon L, Fisher AG, Merkenschlager M (2008). Cohesins functionally associate with CTCF on mammalian chromosome arms. Cell.

[73] Rubio ED, Reiss DJ, Welcsh PL, Disteche CM, Filippova GN, Baliga NS, Aebersold R, Ranish JA, Krumm A (2008). CTCF physically links cohesin to chromatin. Proc Natl Acad Sci U S A.

[74] Barski A, Cuddapah S, Cui K, Roh TY, Schones DE, Wang Z, Wei G, Chepelev I, Zhao K (2007). High-resolution profiling of histone methylations in the human genome. Cell.

[75] Shen Y, Yue F, McCleary DF, Ye Z, Edsall L, Kuan S, Wagner U, Dixon J, Lee L, Lobanenkov VV, Ren B (2012). A map of the *cis*-regulatory sequences in the mouse genome. Nature.

[76] Hsieh CL, Fei T, Chen Y, Li T, Gao Y, Wang X, Sun T, Sweeney CJ, Lee GS, Chen S, Balk SP, Liu XS, Brown M, Kantoff PW (2014). Enhancer RNAs participate in androgen receptor-driven looping that selectively enhances gene activation. Proc Natl Acad Sci U S A.

[77] Hah N, Murakami S, Nagari A, Danko CG, Kraus WL (2013). Enhancer transcripts mark active estrogen receptor binding sites. Genome Res.

[78] Yegnasubramanian S, Wu Z, Haffner MC, Esopi D, Aryee MJ, Badrinath R, He TL, Morgan JD, Carvalho B, Zheng Q, De Marzo AM, Irizarry RA, Nelson WG (2011). Chromosome-wide mapping of DNA methylation patterns in normal and malignant prostate cells reveals pervasive methylation of gene-associated and conserved intergenic sequences. BMC Genomics.

[79] Aran D, Sabato S, Hellman A (2013). DNA methylation of distal regulatory sites characterizes dysregulation of cancer genes. Genome Biol.

[80] Taberlay PC, Statham AL, Kelly TK, Clark SJ, Jones PA (2014). Reconfiguration of nucleosome-depleted regions at distal regulatory elements accompanies DNA methylation of enhancers and insulators in cancer. Genome Res.

[81] Akhtar-Zaidi B, Cowper-Sal-lari R, Corradin O, Saiakhova A, Bartels CF, Balasubramanian D, Myeroff L, Lutterbaugh J, Jarrar A, Kalady MF, Willis J, Moore JH, Tesar PJ, Laframboise T, Markowitz S, Lupien M, Scacheri PC (2012). Epigenomic enhancer profiling defines a signature of colon cancer. Science.

[82] Loven J, Hoke HA, Lin CY, Lau A, Orlando DA, Vakoc CR, Bradner JE, Lee TI, Young RA (2013). Selective inhibition of tumor oncogenes by disruption of super-enhancers. Cell.

[83] Chapuy B, McKeown MR, Lin CY, Monti S, Roemer MG, Qi J, Rahl PB, Sun HH, Yeda KT, Doench JG, Reichert E, Kung AL, Rodig SJ, Young RA, Shipp MA, Bradner JE (2013). Discovery and characterization of super-enhancer-associated dependencies in diffuse large B cell lymphoma. Cancer Cell.

[84] Magnani L, Stoeck A, Zhang X, Lanczky A, Mirabella AC, Wang TL, Gyorffy B, Lupien M (2013). Genome-wide reprogramming of the chromatin landscape underlies endocrine therapy resistance in breast cancer. Proc Natl Acad Sci U S A.

[85] Knoechel B, Roderick JE, Williamson KE, Zhu J, Lohr JG, Cotton MJ, Gillespie SM, Fernandez D, Ku M, Wang H, Piccioni F, Silver SJ, Jain M, Pearson D, Kluk MJ, Ott CJ, Shultz LD, Brehm MA, Greiner DL, Gutierrez A, Stegmaier K, Kung AL, Root DE, Bradner JE, Aster JC, Kelliher MA, Bernstein BE (2014). An epigenetic mechanism of resistance to targeted therapy in T cell acute lymphoblastic leukemia. Nat Genet.

[86] Okano M, Bell DW, Haber DA, Li E (1999). DNA methyltransferases Dnmt3a and Dnmt3b are essential for *de novo* methylation and mammalian development. Cell.

[87] Kogo R, Shimamura T, Mimori K, Kawahara K, Imoto S, Sudo T, Tanaka F, Shibata K, Suzuki A, Komune S, Miyano S, Mori M (2011). Long noncoding RNA HOTAIR regulates polycomb-dependent chromatin modification and is associated with poor prognosis in colorectal cancers. Cancer Res.

[88] Li D, Feng J, Wu T, Wang Y, Sun Y, Ren J, Liu M (2013). Long intergenic noncoding RNA HOTAIR is overexpressed and regulates PTEN methylation in laryngeal squamous cell carcinoma. Am J Pathol.

[89] Quagliata L, Matter MS, Piscuoglio S, Arabi L, Ruiz C, Procino A, Kovac M, Moretti F, Makowska Z, Boldanova T, Andersen JB, Hämmerle M, Tornillo L, Heim MH, Diederichs S, Cillo C, Terracciano LM (2014). Long noncoding RNA HOTTIP/HOXA13 expression is associated with disease progression and predicts outcome in hepatocellular carcinoma patients. Hepatology.

[90] Xiang JF, Yin QF, Chen T, Zhang Y, Zhang XO, Wu Z, Zhang S, Wang HB, Ge J, Lu X, Yang L, Chen LL (2014). Human colorectal cancer-specific CCAT1-L lncRNA regulates long-range chromatin interactions at the *MYC* locus. Cell Res.

[91] Nissan A, Stojadinovic A, Mitrani-Rosenbaum S, Halle D, Grinbaum R, Roistacher M, Bochem A, Dayanc BE, Ritter G, Gomceli I, Bostanci EB, Akoglu M, Chen YT, Old LJ, Gure AO (2012). Colon cancer associated transcript-1: a novel RNA expressed in malignant and pre-malignant human tissues. Int J Cancer.

[92] Dontu G, Jackson KW, McNicholas E, Kawamura MJ, Abdallah WM, Wicha MS (2004). Role of Notch signaling in cell-fate determination of human mammary stem/progenitor cells. Breast Cancer Res.

[93] Claudio JO, Masih-Khan E, Tang H, Goncalves J, Voralia M, Li ZH, Nadeem V, Cukerman E, Francisco-Pabalan O, Liew CC, Woodgett JR, Stewart AK (2002). A molecular compendium of genes expressed in multiple myeloma. Blood.

[94] Carrasco DR, Sukhdeo K, Protopopova M, Sinha R, Enos M, Carrasco DE, Zheng M, Mani M, Henderson J, Pinkus GS, Munshi N, Horner J, Ivanova EV, Protopopov A, Anderson KC, Tonon G, DePinho RA (2007). The differentiation and stress response factor XBP-1 drives multiple myeloma pathogenesis. Cancer Cell.

[95] Schaub MA, Boyle AP, Kundaje A, Batzoglou S, Snyder M (2012). Linking disease associations with regulatory information in the human genome. Genome Res.

[96] Cowper-Sal lari R, Zhang X, Wright JB, Bailey SD, Cole MD, Eeckhoute J, Moore JH, Lupien M (2012). Breast cancer risk-associated SNPs modulate the affinity of chromatin for FOXA1 and alter gene expression. Nat Genetics.

[97] Ernst J, Kheradpour P, Mikkelsen TS, Shoresh N, Ward LD, Epstein CB, Zhang X, Wang L, Issner R, Coyne M, Ku M, Durham T, Kellis M, Bernstein BE (2011). Mapping and analysis of chromatin state dynamics in nine human cell types. Nature.

[98] Maurano MT, Humbert R, Rynes E, Thurman RE, Haugen E, Wang H, Reynolds AP, Sandstrom R, Qu H, Brody J, Shafer A, Neri F, Lee K, Kutyavin T, Stehling-Sun S, Johnson AK, Canfield TK, Giste E, Diegel M, Bates D, Hansen RS, Neph S, Sabo PJ, Heimfeld S, Raubitschek A, Ziegler S, Cotsapas C, Sotoodehnia N, Glass I, Sunyaev SR (2012). Systematic localization of common disease-associated variation in regulatory DNA. Science.

[99] Ahmadiyeh N, Pomerantz MM, Grisanzio C, Herman P, Jia L, Almendro V, He HH, Brown M, Liu XS, Davis M, Caswell JL, Beckwith CA, Hills A, Macconaill L, Coetzee GA, Regan MM, Freedman ML (2010). 8q24 prostate, breast, and colon cancer risk loci show tissue-specific long-range interaction with MYC. Proc Natl Acad Sci U S A.

[100] Tuupanen S, Turunen M, Lehtonen R, Hallikas O, Vanharanta S, Kivioja T, Bjorklund M, Wei G, Yan J, Niittymäki I, Mecklin JP, Järvinen H, Ristimäki A, Di-Bernardo M, East P, Carvajal-Carmona L, Houlston RS, Tomlinson I, Palin K, Ukkonen E, Karhu A, Taipale J, Aaltonen LA (2009). The common colorectal cancer predisposition SNP rs6983267 at chromosome 8q24 confers potential to enhanced Wnt signaling. Nat Genet.

[101] Wright JB, Brown SJ, Cole MD (2010). Upregulation of c-*MYC* in *cis* through a large chromatin loop linked to a cancer risk-associated single-nucleotide polymorphism in colorectal cancer cells. Mol Cell Biol.

[102] Zhang X, Bailey SD, Lupien M (2014). Laying a solid foundation for Manhattan - 'setting the functional basis for the post-GWAS era'. Trends Genet.

[103] Sotelo J, Esposito D, Duhagon MA, Banfield K, Mehalko J, Liao H, Stephens RM, Harris TJ, Munroe DJ, Wu X (2010). Long-range enhancers on 8q24 regulate c-Myc. Proc Natl Acad Sci U S A.

[104] Steidl U, Steidl C, Ebralidze A, Chapuy B, Han HJ, Will B, Rosenbauer F, Becker A, Wagner K, Koschmieder S, Kobayashi S, Costa DB, Schulz T, O'Brien KB, Verhaak RG, Delwel R, Haase D, Trümper L, Krauter J, Kohwi-Shigematsu T, Griesinger F, Tenen DG (2007). A distal single nucleotide polymorphism alters long-range regulation of the *PU.1* gene in acute myeloid leukemia. J Clin Invest.

[105] Aran D, Hellman A (2013). DNA methylation of transcriptional enhancers and cancer predisposition. Cell.

[106] Chapman MA, Lawrence MS, Keats JJ, Cibulskis K, Sougnez C, Schinzel AC, Harview CL, Brunet JP, Ahmann GJ, Adli M, Anderson KC, Ardlie KG, Auclair D, Baker A, Bergsagel PL, Bernstein BE, Drier Y, Fonseca R, Gabriel SB, Hofmeister CC, Jagannath S, Jakubowiak AJ, Krishnan A, Levy J, Liefeld T, Lonial S, Mahan S, Mfuko B, Monti S, Perkins LM (2011). Initial genome sequencing and analysis of multiple myeloma. Nature.

[107] Pleasance ED, Stephens PJ, O'Meara S, McBride DJ, Meynert A, Jones D, Lin ML, Beare D, Lau KW, Greenman C, Varela I, Nik-Zainal S, Davies HR, Ordoñez GR, Mudie LJ, Latimer C, Edkins S, Stebbings L, Chen L, Jia M, Leroy C, Marshall J, Menzies A, Butler A, Teague JW, Mangion J, Sun YA, McLaughlin SF, Peckham HE, Tsung EF (2010). A small-cell lung cancer genome with complex signatures of tobacco exposure. Nature.

[108] Wang K, Yuen ST, Xu J, Lee SP, Yan HH, Shi ST, Siu HC, Deng S, Chu KM, Law S, Chan KH, Chan AS, Tsui WY, Ho SL, Chan AK, Man JL, Foglizzo V, Ng MK, Chan AS, Ching YP, Cheng GH, Xie T, Fernandez J, Li VS, Clevers H, Rejto PA, Mao M, Leung S (2014). Whole-genome sequencing and comprehensive molecular profiling identify new driver mutations in gastric cancer. Nat Genet.

[109] Dalla-Favera R, Bregni M, Erikson J, Patterson D, Gallo RC, Croce CM (1982). Human *c-myc onc* gene is located on the region of chromosome 8 that is translocated in Burkitt lymphoma cells. Proc Natl Acad Sci U S A.

[110] Park SS, Kim JS, Tessarollo L, Owens JD, Peng L, Han SS, Tae Chung S, Torrey TA, Cheung WC, Polakiewicz RD, McNeil N, Ried T, Mushinski JF, Morse HC, Janz S (2005). Insertion of c-*Myc* into *Igh* induces B-cell and plasma-cell neoplasms in mice. Cancer Res.

[111] Koenig SC, Becirevic E, Hellberg MS, Li MY, So JC, Hankins JS, Ware RE, McMahon L, Steinberg MH, Luo HY, Chui DH (2009). Sickle cell disease caused by heterozygosity for Hb S and novel LCR deletion: report of two patients. Am J Hematol.

[112] Goutagny S, Nault JC, Mallet M, Henin D, Rossi JZ, Kalamarides M (2014). High incidence of activating TERT promoter mutations in meningiomas undergoing malignant progression. Brain Pathol.

[113] Horn S, Figl A, Rachakonda PS, Fischer C, Sucker A, Gast A, Kadel S, Moll I, Nagore E, Hemminki K, Schadendorf D, Kumar R (2013). TERT promoter mutations in familial and sporadic melanoma. Science.

[114] Huang FW, Hodis E, Xu MJ, Kryukov GV, Chin L, Garraway LA (2013). Highly recurrent TERT promoter mutations in human melanoma. Science.

[115] Liu X, Bishop J, Shan Y, Pai S, Liu D, Murugan AK, Sun H, El-Naggar AK, Xing M (2013). Highly prevalent TERT promoter mutations in aggressive thyroid cancers. Endocr Relat Cancer.

[116] Rachakonda PS, Hosen I, de Verdier PJ, Fallah M, Heidenreich B, Ryk C, Wiklund NP, Steineck G, Schadendorf D, Hemminki K, Kumar R (2013). TERT promoter mutations in bladder cancer affect patient survival and disease recurrence through modification by a common polymorphism. Proc Natl Acad Sci U S A.

[117] Gordon CT, Tan TY, Benko S, Fitzpatrick D, Lyonnet S, Farlie PG (2009). Long-range regulation at the *SOX9* locus in development and disease. J Med Genet.

[118] Gurnett CA, Bowcock AM, Dietz FR, Morcuende JA, Murray JC, Dobbs MB (2007). Two novel point mutations in the long-range SHH enhancer in three families with triphalangeal thumb and preaxial polydactyly. Am J Med Genet A.

[119] Smemo S, Campos LC, Moskowitz IP, Krieger JE, Pereira AC, Nobrega MA (2012). Regulatory variation in a TBX5 enhancer leads to isolated congenital heart disease. Hum Mol Genet.

[120] Weedon MN, Cebola I, Patch AM, Flanagan SE, De Franco E, Caswell R, Rodriguez-Segui SA, Shaw-Smith C, Cho CH, Lango Allen H, Houghton JA, Roth CL, Chen R, Hussain K, Marsh P, Vallier L, Murray A, Ellard S, Ferrer J, Hattersley AT, International Pancreatic Agenesis Consortium (2014). Recessive mutations in a distal PTF1A enhancer cause isolated pancreatic agenesis. Nat Genet.

[121] Barbieri CE, Baca SC, Lawrence MS, Demichelis F, Blattner M, Theurillat JP, White TA, Stojanov P, Van Allen E, Stransky N, Nickerson E, Chae SS, Boysen G, Auclair D, Onofrio RC, Park K, Kitabayashi N, MacDonald TY, Sheikh K, Vuong T, Guiducci C, Cibulskis K, Sivachenko A, Carter SL, Saksena G, Voet D, Hussain WM, Ramos AH, Winckler W, Redman MC (2012). Exome sequencing identifies recurrent SPOP, FOXA1 and MED12 mutations in prostate cancer. Nat Genet.

[122] Kandoth C, McLellan MD, Vandin F, Ye K, Niu B, Lu C, Xie M, Zhang Q, McMichael JF, Wyczalkowski MA, Leiserson MD, Miller CA, Welch JS, Walter MJ, Wendl MC, Ley TJ, Wilson RK, Raphael BJ, Ding L (2013). Mutational landscape and significance across 12 major cancer types. Nature.

[123] Pasquet M, Bellanne-Chantelot C, Tavitian S, Prade N, Beaupain B, Larochelle O, Petit A, Rohrlich P, Ferrand C, Van Den Neste E, Poirel HA, Lamy T, Ouachée-Chardin M, Mansat-De Mas V, Corre J, Récher C, Plat G, Bachelerie F, Donadieu J, Delabesse E (2013). High frequency of GATA2 mutations in patients with mild chronic neutropenia evolving to MonoMac syndrome, myelodysplasia, and acute myeloid leukemia. Blood.

[124] Gaynor KU, Grigorieva IV, Allen MD, Esapa CT, Head RA, Gopinath P, Christie PT, Nesbit MA, Jones JL, Thakker RV (2013). GATA3 mutations found in breast cancers may be associated with aberrant nuclear localization, reduced transactivation and cell invasiveness. Horm Cancer.

[125] Jiang YZ, Yu KD, Zuo WJ, Peng WT, Shao ZM (2014). GATA3 mutations define a unique subtype of luminal-like breast cancer with improved survival. Cancer.

[126] Usary J, Llaca V, Karaca G, Presswala S, Karaca M, He X, Langerød A, Kåresen R, Oh DS, Dressler LG, Lønning PE, Strausberg RL, Chanock S, Børresen-Dale AL, Perou CM (2004). Mutation of GATA3 in human breast tumors. Oncogene.

[127] Lawrence MS, Stojanov P, Mermel CH, Robinson JT, Garraway LA, Golub TR, Meyerson M, Gabriel SB, Lander ES, Getz G (2014). Discovery and saturation analysis of cancer genes across 21 tumour types. Nature.

[128] Lam K, Zhang DE (2012). RUNX1 and RUNX1-ETO: roles in hematopoiesis and leukemogenesis. Front Biosci.

[129] Herz HM, Hu D, Shilatifard A (2014). Enhancer malfunction in cancer. Mol Cell.

[130] Morin RD, Johnson NA, Severson TM, Mungall AJ, An J, Goya R, Paul JE, Boyle M, Woolcock BW, Kuchenbauer F, Yap D, Humphries RK, Griffith OL, Shah S, Zhu H, Kimbara M, Shashkin P, Charlot JF, Tcherpakov M, Corbett R, Tam A, Varhol R, Smailus D, Moksa M, Zhao Y, Delaney A, Qian H, Birol I, Schein J, Moore R (2010). Somatic mutations altering EZH2 (Tyr641) in follicular and diffuse large B-cell lymphomas of germinal-center origin. Nat Genet.

[131] Ley TJ, Ding L, Walter MJ, McLellan MD, Lamprecht T, Larson DE, Kandoth C, Payton JE, Baty J, Welch J, Harris CC, Lichti CF, Townsend RR, Fulton RS, Dooling DJ, Koboldt DC, Schmidt H, Zhang Q, Osborne JR, Lin L, O'Laughlin M, McMichael JF, Delehaunty KD, McGrath SD, Fulton LA, Magrini VJ, Vickery TL, Hundal J, Cook LL, Conyers JJ (2010). DNMT3A mutations in acute myeloid leukemia. N Engl J Med.

[132] Delhommeau F, Dupont S, Della Valle V, James C, Trannoy S, Massé A, Kosmider O, Le Couedic JP, Robert F, Alberdi A, Lécluse Y, Plo I, Dreyfus FJ, Marzac C, Casadevall N, Lacombe C, Romana SP, Dessen P, Soulier J, Viguié F, Fontenay M, Vainchenker W, Bernard OA (2009). Mutation in TET2 in myeloid cancers. N Engl J Med.

[133] Filippova GN, Qi CF, Ulmer JE, Moore JM, Ward MD, Hu YJ, Loukinov DI, Pugacheva EM, Klenova EM, Grundy PE, Feinberg AP, Cleton-Jansen AM, Moerland EW, Cornelisse CJ, Suzuki H, Komiya A, Lindblom A, Dorion-Bonnet F, Neiman PE, Morse HC, Collins SJ, Lobanenkov VV (2002). Tumor-associated zinc finger mutations in the CTCF transcription factor selectively alter tts DNA-binding specificity. Cancer Res.

[134] Solomon DA, Kim JS, Bondaruk J, Shariat SF, Wang ZF, Elkahloun AG, Ozawa T, Gerard J, Zhuang D, Zhang S, Navai N, Siefker-Radtke A, Phillips JJ, Robinson BD, Rubin MA, Volkmer B, Hautmann R, Küfer R, Hogendoorn PC, Netto G, Theodorescu D, James CD, Czerniak B, Miettinen M, Waldman T (2013). Frequent truncating mutations of STAG2 in bladder cancer. Nat Genet.

[135] Guo G, Sun X, Chen C, Wu S, Huang P, Li Z, Dean M, Huang Y, Jia W, Zhou Q, Tang A, Yang Z, Li X, Song P, Zhao X, Ye R, Zhang S, Lin Z, Qi M, Wan S, Xie L, Fan F, Nickerson ML, Zou X, Hu X, Xing L, Lv Z, Mei H, Gao S, Liang C (2013). Whole-genome and whole-exome sequencing of bladder cancer identifies frequent alterations in genes involved in sister chromatid cohesion and segregation. Nat Genet.

[136] Assie G, Letouze E, Fassnacht M, Jouinot A, Luscap W, Barreau O, Omeiri H, Rodriguez S, Perlemoine K, Rene-Corail F, Elarouci N, Sbiera S, Kroiss M, Allolio B, Waldmann J, Quinkler M, Mannelli M, Mantero F, Papathomas T, De Krijger R, Tabarin A, Kerlan V, Baudin E, Tissier F, Dousset B, Groussin L, Amar L, Clauser E, Bertagna X, Ragazzon B (2014). Integrated genomic characterization of adrenocortical carcinoma. Nat Genet.

[137] Makinen N, Mehine M, Tolvanen J, Kaasinen E, Li Y, Lehtonen HJ, Gentile M, Yan J, Enge M, Taipale M, Aavikko M, Katainen R, Virolainen E, Böhling T, Koski TA, Launonen V, Sjöberg J, Taipale J, Vahteristo P, Aaltonen LA (2011). MED12, the mediator complex subunit 12 gene, is mutated at high frequency in uterine leiomyomas. Science.

[138] Robinson JL, Holmes KA, Carroll JS (2013). FOXA1 mutations in hormone-dependent cancers. Front Oncol.

[139] Tsai FY, Keller G, Kuo FC, Weiss M, Chen J, Rosenblatt M, Alt FW, Orkin SH (1994). An early haematopoietic defect in mice lacking the transcription factor GATA-2. Nature.

[140] Tsai FY, Orkin SH (1997). Transcription factor GATA-2 is required for proliferation/survival of early hematopoietic cells and mast cell formation, but not for erythroid and myeloid terminal differentiation. Blood.

[141] Kouros-Mehr H, Slorach EM, Sternlicht MD, Werb Z (2006). GATA-3 maintains the differentiation of the luminal cell fate in the mammary gland. Cell.

[142] Ito S, D'Alessio AC, Taranova OV, Hong K, Sowers LC, Zhang Y (2010). Role of Tet proteins in 5mC to 5hmC conversion, ES-cell self-renewal and inner cell mass specification. Nature.

[143] Gupta RA, Shah N, Wang KC, Kim J, Horlings HM, Wong DJ, Tsai MC, Hung T, Argani P, Rinn JL, Wang Y, Brzoska P, Kong B, Li R, West RB, van de Vijver MJ, Sukumar S, Chang HY (2010). Long non-coding RNA HOTAIR reprograms chromatin state to promote cancer metastasis. Nature.

[144] Yang L, Lin C, Jin C, Yang JC, Tanasa B, Li W, Merkurjev D, Ohgi KA, Meng D, Zhang J, Evans CP, Rosenfeld MG (2013). lncRNA-dependent mechanisms of androgen-receptor-regulated gene activation programs. Nature.

[145] Prensner JR, Sahu A, Iyer MK, Malik R, Chandler B, Asangani IA, Poliakov A, Vergara IA, Alshalalfa M, Jenkins RB, Davicioni E, Feng FY, Chinnaiyan AM (2014). The lncRNAs *PCGEM1* and *PRNCR1* are not implicated in castration resistant prostate cancer. Oncotarget.

[146] Bae JB (2013). Perspectives of international human epigenome consortium. Genomics Inform.

[147] Bernstein BE, Stamatoyannopoulos JA, Costello JF, Ren B, Milosavljevic A, Meissner A, Kellis M, Marra MA, Beaudet AL, Ecker JR, Farnham PJ, Hirst M, Lander ES, Mikkelsen TS, Thomson JA (2010). The NIH roadmap epigenomics mapping consortium. Nat Biotechnol.

[148] Meacham CE, Morrison SJ (2013). Tumour heterogeneity and cancer cell plasticity. Nature.

[149] Lim E, Vaillant F, Wu D, Forrest NC, Pal B, Hart AH, Asselin-Labat ML, Gyorki DE, Ward T, Partanen A, Feleppa F, Huschtscha LI, Thorne HJ, Fox SB, Yan M, French JD, Brown MA, Smyth GK, Visvader JE, Lindeman GJ, kConFab (2009). Aberrant luminal progenitors as the candidate target population for basal tumor development in BRCA1 mutation carriers. Nat Med.

[150] Melchor L, Molyneux G, Mackay A, Magnay FA, Atienza M, Kendrick H, Nava-Rodrigues D, López-García MÁ, Milanezi F, Greenow K, Robertson D, Palacios J, Reis-Filho JS, Smalley MJ (2014). Identification of cellular and genetic drivers of breast cancer heterogeneity in genetically engineered mouse tumour models. J Pathol.

[151] Molyneux G, Geyer FC, Magnay FA, McCarthy A, Kendrick H, Natrajan R, Mackay A, Grigoriadis A, Tutt A, Ashworth A, Reis-Filho JS, Smalley MJ (2010). BRCA1 basal-like breast cancers originate from luminal epithelial progenitors and not from basal stem cells. Cell Stem Cell.

[152] Proia TA, Keller PJ, Gupta PB, Klebba I, Jones AD, Sedic M, Gilmore H, Tung N, Naber SP, Schnitt S, Lander ES, Kuperwasser C (2011). Genetic predisposition directs breast cancer phenotype by dictating progenitor cell fate. Cell Stem Cell.

